# Recent immigration raids increased student absences

**DOI:** 10.1073/pnas.2510395122

**Published:** 2025-11-04

**Authors:** Thomas S. Dee

**Affiliations:** ^a^Graduate School of Education, Stanford University, Stanford, CA 94305; ^b^Hoover Institution, Stanford University, Stanford, CA 94305; ^c^Stanford Institute for Economic Policy Research, Stanford University, Stanford, CA 94305

**Keywords:** immigration, education, public policy

## Abstract

Local immigration raids expanded dramatically across the United States during the first 2 mo of 2025. Anecdotal accounts suggest that these raids increased student absences from schools because parents fear being separated from their children. This study evaluates this claim using a daily time series of school absences spanning the current and two prior school years from five school districts serving communities subject to recent and unexpected raids in California’s Central Valley. The results indicate that recent raids coincided with a 22 percent increase in daily student absences with particularly large increases among the youngest students. These increased absences underscore the broader policy relevance of this immigration enforcement in terms of their impact on schools, childhood stress, and opportunities to learn.

Current estimates indicate nearly 80 percent of the foreign-born individuals in the United States are legal residents. The remaining group of unauthorized immigrants—an estimated 11 million individuals—constitute 3.3 percent of the total US population (1). The design and enforcement of laws and regulations targeting these unauthorized immigrants are an active and highly contested domain of public policy in the United States. Much of the research relevant to this debate has focused on whether federal initiatives that create immigration-enforcement partnerships with state and local authorities (e.g., Secure Communities and 287(g) agreements) influence outcomes such as population mobility, crime, and economic activity (2–4).

A substantial amount of research has also focused on how interior immigration enforcement impacts different dimensions of child development (5). Over 5 million children under age 18 live with a parent who is an unauthorized immigrant, and the vast majority of these children are US citizens (1). The persistent threat that a parent may be jailed and deported can attenuate the learning opportunities available to these children through, for example, its pejorative effects on childhood stress and economic opportunity. The developmental implications of a social climate of fear and mistrust may also extend to other children such as those connected to legal immigrants, those who have demographic identities associated with immigrant status (e.g., Hispanic ethnicity), and their peers (6). Immigration enforcement may also have broader effects through its impact on schools and teachers seeking to support affected students and manage instructional pacing. Multiple studies conclude that prior instances of interior immigration enforcement have had negative effects on a diverse array of child outcomes such as grade retention, high-school completion, test scores, and anxiety disorders (7–9).

Several studies have also focused on student absences as both a highly relevant educational outcome and as a leading indicator of the potential downstream effects of immigration enforcement for youth development. However, the evidence on the distinctive impact of immigration raids on student absences is more limited and mixed. Relative to structured immigration-enforcement initiatives, raids may have larger effects because they are comparatively sharp, unanticipated, and highly localized or smaller effects because they are seen as transitory events. Two prior studies indicate that immigration raids and arrests increase student absences (8, 9). However, another study suggests that spikes in relevant arrests (i.e., a proxy for raids) do not clearly influence student absences because the serious threats they imply are a constant factor in the decisions made in families with undocumented members (10).

This study makes two contributions by examining a prominent immigration raid coinciding with the beginning of the current Presidential administration. First, it adds to the limited findings that focus specifically on immigration raids rather than other more programmatic forms of interior immigration enforcement. Second, this study provides leading evidence on a distinctive—and ongoing—change in immigration enforcement. An unusually broad and sharp increase in immigration raids and arrests characterized the beginning of the second Trump administration (11). The Administration’s decision to rescind a 2011 directive that prohibited immigration enforcement in “sensitive areas” such as schools and houses of worship amplified the threats implied by this increased enforcement (12).

The specific context for this study is California’s Central Valley, a prominent agricultural region with a substantial resident population of immigrants. An unanticipated enforcement effort by US Customs and Border Patrol (CBP), named “Operation Return to Sender,” began in this area on January 7, 2025 (i.e., the day after the 2024 Presidential election was certified by the US Congress). Officials from the Biden Administration claimed a CBP agent who “went rogue” directed the operation without informing other officials (13). The CBP subsequently stated it conducted a 4-d “targeted enforcement” focused on unauthorized immigrants with criminal records that resulted in 78 arrests. However, other observers characterized the effort as a broad dragnet targeted in locations frequented by immigrant workers and resulting in roughly 1,000 detentions (14).

Subsequent accounts noted that these raids “sent shock waves across the Central Valley, where a largely immigrant workforce helps harvest a quarter of the food grown in the U.S.” (13). The public schools in this southern region of California’s Central Valley (i.e., Kern, Kings, Tulare, and Fresno counties) serve over half a million students, more than 70 percent of whom are Hispanic. Anecdotal accounts suggested that student absences increased in the region as families expressed fear over sending their children to school (15). This study provides evidence on this question by examining 3 y of daily data on student absences in five Central Valley school districts.

## Methods and Materials

These data identify the daily count of student absences in each district during the 2022–23 and 2023–24 schools as well as the 2024–25 school year through February (n = 2,234 district-by-day observations). These daily counts across 3 y make it possible to evaluate whether student absences during the recent increase in immigration enforcement differed from what would be expected based on the seasonal patterns observed within prior school years.

Specifically, I examine ordinary least squares regression specifications where the dependent variable, the natural logarithm of student absences, is a function of fixed effects unique to each district, to each academic year, each month, and each day of the week (see *SI Appendix* for further details). The independent variable of interest is a binary indicator equal to 1 only on days after the raid began. This research design effectively identifies whether the number of student absences during the recent immigration raids (i.e., January and February of 2025) is distinctly different from the within-year, seasonal patterns observed previously. Additional specifications introduce controls for other attendance-related events and allow the year, month, and day fixed effects to vary by district. An event-study version of this fixed-effect design identifies effects unique to each month during the 2024–25 school year relative to the within-year patterns established in the two prior school years.

## Results

**Fig. 1.**
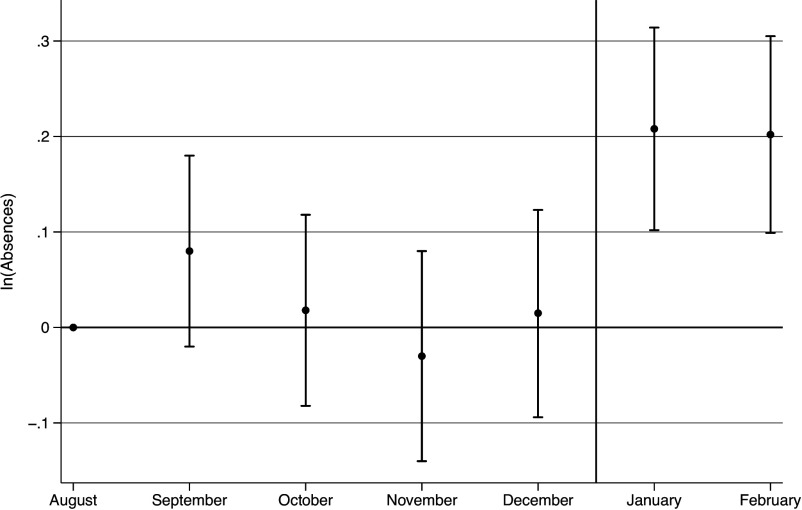
Event-study estimates, student absences by month during the 2024–25 school year.

Fig. 1 presents the key event-study results, which provide an initial and unrestrictive approach to assessing the impact of the recent immigration raids on student absences. These results indicate a sharp increase of over 20 percent in student absences during the immigration-raid period. An F-test rejects the null hypothesis that the increased absences observed in January and February of 2025 jointly equal zero (*P* = 0.0030). The event-study results also indicate the impact of the raids persisted through the study period. An F-test cannot reject the null hypothesis that effects observed in January and February of 2025 are equal (*P* = 0.8565)

Two other aspects of the event-study evidence in Fig. 1 merit emphasis. First, the evidence that the increased absences are similarly high in February (i.e., weeks after the initial raid) indicates that these effects were not transitory over this period. Second, the monthly patterns of student absences in the fall of 2024 (i.e., just before the immigration raids) provide evidence consistent with the internal validity of the research design. That is, the absence counts in the months just prior to the immigration raids resemble the monthly patterns observed within prior school years. Specifically, t-tests do not reject the null hypotheses that the estimated effects unique to each month in Fall 2024 are zero. This lack of distinctive preraid trends is consistent with the maintained assumption that there are not empirically relevant and unobserved confounds in the 2024–25 school year when the recent immigration raids occurred.

Table 1 presents direct estimates of the impact of the immigration raids on student absences across three different specifications. The first specification conditions on fixed effects unique to each district, each day of the week, each month, and each school year. The second specification adds three binary indicators identifying attendance-relevant events. The third specification introduces fixed effects unique to each district-year interaction, each district-month interaction and district-specific fixed effects unique to each day of the week. These results consistently indicate that the immigration raids implied a large and statistically significant increase in the natural log of student absences ($\hat{\beta }$ = 0.195, *P* < 0.0001). This estimate implies that the recent immigration raids increased the count of daily student absences by 22 percent (i.e., $e^{0.195}-1$).

**Table 1.** Estimated effects of immigration raids on student absences

Student group	(1)	(2)	(3)
All	0.220*** (0.0238)	0.194*** (0.0196)	0.195*** (0.0179)
Prekindergarten	0.319*** (0.0460)	0.299*** (0.0449)	0.303*** (0.0436)
Grades K to 5	0.290*** (0.0298)	0.268*** (0.0262)	0.269*** (0.0242)
Grades 6 to 8	0.199*** (0.0314)	0.268*** (0.0262)	0.169*** (0.0245)
Grades 9 to 12	0.0997** (0.0428)	0.0771* (0.0393)	0.0777** (0.0363)
Event FE	No	Yes	Yes
District-Year FE	No	No	Yes
District-Month FE	No	No	Yes
District-Day of Week FE	No	No	Yes

Notes: The data consist of daily observations of student absences in five school districts over the 2022–23 and 2023–24 schools and the 2024–25 school year through February (n = 2,234). The dependent variable is the natural log of absences and all specifications condition on fixed effects unique to each district, each school year, each month, and each day of the week. A binary indicator identifies the period of immigration raids. Robust SE are reported in parentheses. See *SI Appendix* for further details.

***P < 0.01, **P < 0.05, *P < 0.1.

Both the robustness of this estimated effect across the different specifications in Table 1 and the event-study evidence (Fig. 1) suggest the reliability of this result. Three other sets of results underscore the robustness of these results. First, I find similar results across alternative approaches to estimation and inference that include bootstrapping and Poisson and negative-binomial regressions that explicitly accommodate the count nature of the dependent variable. Second, I also find the effects estimated separately for each of the 5 districts are consistently large, positive, and statistically significant. Third, applying this research design to similar data available from a Riverside County school district located over 200 hundred miles from the raid results in a smaller and statistically insignificant estimate ($\hat{\beta }$ = 0.057, *P* = 0.269).

Table 1 also reports the estimated effects of the immigration raids on the daily count of student absences defined separately for students in four different grade spans (i.e., Pre-kindergarten, grades K to 5, grades 6 to 8, and grades 9 to 12). Immigration raids may have larger effects on absences among younger children for at least two reasons. One is that, relative to other US children, those living with undocumented immigrants tend to be younger. Second, undocumented immigrants may be especially concerned about being separated in response to an immigration raid when their family includes younger children. The results in Table 1 are consistent with this expectation. The estimated effects of immigration raids on the daily counts of student absences are positive and statistically significant in each grade span as well as consistent across specifications. However, the estimated effect among students in grades K to 5 ($\hat{\beta }$ = 0.269, *P* < 0.0001) is over three times larger than the estimated effect among students in high school ($\hat{\beta }$ = 0.078, *P* = 0.032).

## Discussion

Using unique data from school districts in California’s Central Valley, this study presents leading evidence that the recent surge in interior immigration enforcement significantly increased student absences from school. Specifically, these results indicate these raids caused a 22-percent increase in daily counts of student absences. These increases were particularly sharp among younger students and have endured over the 2-mo period for which postraid data are currently available. Another way to frame this impact is to note that, in the 2023–24 school year, the public schools in this four-county region served roughly 537,000 students. On average, these students were absent from school on 12.4 d. A 22-percent increase in such absences implies 1.4 additional absences per student over half a school year. In the aggregate, this increase implies over 725,000 student days lost in the four-county region due to the raids.

However, the impact of immigration raids on student absences has policy relevance beyond its implications for school absences among directly affected students. For example, there may be educational implications for all students as the pacing and character of classroom instruction respond to the pedagogical challenges created by increased absenteeism and stress among students. Increased absenteeism also adds to the academic-recovery challenges faced by schools and districts in the wake of the COVID-19 pandemic. Those pre-existing challenges include the sharp and largely enduring increases in chronic absenteeism as schools returned to traditional in-person instruction. The increased absenteeism due to immigration raids may also presage enrollment losses that add to the fiscal challenges faced by school districts after the pandemic flight from public schools. Immigration raids may also reduce school resources in states like California where state aid is based in part on average daily attendance. Critically, the substantive implications of these increased absences are also likely to extend well beyond educational settings. The increased absences can also be understood as a leading indicator of broad and developmentally harmful stress these raids create for students and their families. However, whether the most recent immigration raids have these effects and do so beyond the California’s Central Valley is an open question. These issues underscore the need for continued attention to studying these immigration-related policy choices and promoting awareness of evidence on their implications.

## Supplementary Material

Appendix 01 (PDF)

## Data Availability

Stata dataset and code data have been deposited in Stanford Digital Repository (16).
