# Supplementary material for: Recent immigration raids increased student absences
Source: Proc Natl Acad Sci U S A. 2025 Nov 4;122(45):e2510395122. doi: 10.1073/pnas.2510395122 (PMC12625821; doi:10.1073/pnas.2510395122)
Supplement: Supplementary file 1 — Appendix 01 (PDF) [file pnas.2510395122.sapp.pdf]

PNAS

Supporting Information for

Recent Immigration Raids Increased Student Absences

Thomas S. Dee[1,2,3]*

[1]Graduate School of Education, Stanford University, Stanford, CA 94305, US
[2]Hoover Institution, Stanford University, Stanford, CA 94305, US
[3]Stanford Institute for Economic Policy Research (SIEPR), Stanford University, Stanford, CA 94305, US
*Corresponding author.

Email: tdee@stanford.edu.

This PDF file includes:

Materials and Methods

Materials and Methods

Materials

The data for this study consist of a daily time series of all student absences (i.e., both excused and unexcused) for 5 school districts in the southern portion of California's Central Valley over the 2022-23 and 2023-24 school years as well as during the 2024-25 school year through February 2025. Big Local News at Stanford University and I collected these data directly through outreach and public-records requests to these school districts. Because these district-level data are publicly accessible and not individually identified, this study is not defined as human-subject research or subject to oversight by an Institutional Review Board (IRB). To avoid any unintended consequences for these districts of providing these data, they are not identified here. The data and code for the results reported here are publicly available at the Stanford Digital Repository: https://doi.org/10.25740/ps356xc8424.

I received these data from the districts in varied formats. After organizing them into daily counts of student absences, I examined them for outliers and cross-validated them with respect to the corresponding academic calendars that identified valid attendance days for each district and school year. I resolved a small number of inconsistencies, with the support of Big Local News, through follow-up communication with school districts (e.g., shifts in valid attendance days due to weather-related closures and make-up days). I also note that one district only provided data through February for each of the three school years (i.e., excluding March-June). I also excluded days in June from this analysis as it consists of just a few school days, which tend to have idiosyncratically high counts of absenteeism.

The all-student analytical sample consists of 2,234 district-by-day daily counts of student absences over these five school districts and three school years. In 4 districts, the data also allowed the identification of absence counts by the grades served within the district: Pre-K, Elementary (grades K-5), Middle (grades 6-8), and High (grades 9-12). This implies an analytical sample of 1,770 district-by-day data observations. However, the sample of high-school absences consists of 1,290 district-by-day observations from three school districts as one of these school districts does not serve grades 9 through 12.

The independent variable of interest is a binary indicator equal to one for school attendance days on January 8, 2025 (i.e., the day after Operation Return to Sender began) and later. Over 7 percent of the data are observed in this treatment period. The covariates used in the research design below include binary indicators unique to each school district, to each academic year, to each month, and to each day of the week. Binary indicators unique to district-year interaction, each district-month interaction, and each interaction between a district and a day of the week are also included in saturated regression specifications described below.

The covariate set also includes three binary indicators that identify events relevant to school attendance. One is an indicator that identifies the last school day before one of the three major school breaks (i.e., Thanksgiving, winter holidays, and spring holidays). This indicator is defined uniquely for each school district and year based on published calendars. The covariate set also includes a binary indicator for several community-relevant holidays that sometimes overlap with valid school-attendance days. These consist of September 16 (Mexican Independence Day), November 1 and 2 (Day of the Dead), December 12 (Feast Day for Our Lady of Guadalupe), and the first Monday in February (Constitution Day in Mexico). A final covariate is a binary indicator for February 3, 2025. On this day, a “Day without Immigrants” protest occurred and student absences were unusually high. While these protests (and corresponding absences) can be attributed to Operation Return to Sender, it can also be understood as a possible confound when identifying how school absenteeism responded to the threat of arrest.

Methods

The general regression specification used to identify the effects of the recent immigration raids takes a linear form where the dependent variable is the natural logarithm of the count of student absences on day *i* in school district *s* on day of the week *w*, in month *m* and in school year *t*. This

specification conditions on fixed effects unique to each school district, each day of the week, each month, and each school year, respectively. The independent variable of interest identifies days after Operation Return to Sender began (i.e., January 8, 2025 and later). The event-study estimates (Fig. 1) generalize this specification by allowing for fixed effects unique to each month of the 2024-25 school year in lieu of this “treatment” indicator.

The second specification in Table 1 also conditions on the three binary indicators described above that identify days associated with attendance-relevant events (e.g., the day before a major holiday break). The third specification in Table 1 saturates this specification with unrestricted fixed effects unique to each district-year interaction, to each district-month interaction, and to each district interaction with the day of the week.

The baseline results (Table 1) are based on ordinary least-squares estimation and heteroscedastic-consistent standard errors. Alternative approaches to estimation and inference generate similar results. This includes bootstrapping the estimated coefficient on the treatment indicator as well as randomization inference. Four different maximum-likelihood approaches that explicitly recognize the count-data nature of the daily absence counts also generate similar results. These consist of Poisson and negative-binomial regressions that condition on the full covariate set directly as well as conditional maximum-likelihood versions of these count-data regressions that condition on district fixed effects indirectly.
